# Supplementary material for: Adsorption and Desorption Behaviour of Polychlorinated Biphenyls onto Microplastics’ Surfaces in Water/Sediment Systems
Source: Toxics. 2020 Aug 17;8(3):59. doi: 10.3390/toxics8030059 (PMC7560274; doi:10.3390/toxics8030059)
Supplement: Supplementary file 1 [file toxics-08-00059-s001.pdf]

toxics

Supplementary Material: Adsorption and Desorption Behaviour of Polychlorinated Biphenyls onto Microplastics' Surfaces in Water/Sediment Systems

Marta Llorca, Manuela Ábalos, Albert Vega-Herrera, Miquel A. Adrados, Esteban Abad and Marinella Farré *

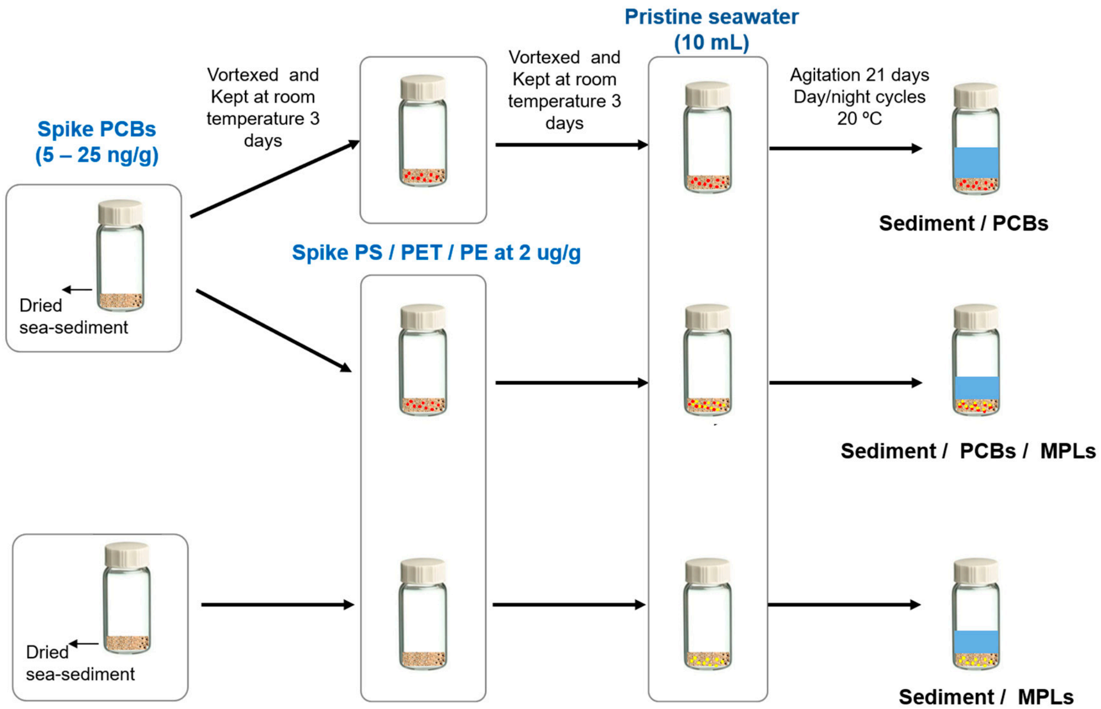

Figure S1. schematic flowchart of experimental design.

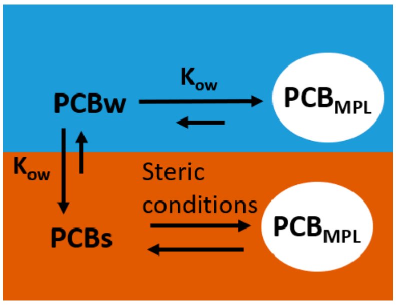

Figure S2. Schematic diagram on sediment, water, and MPLs partitioning for PCBs.

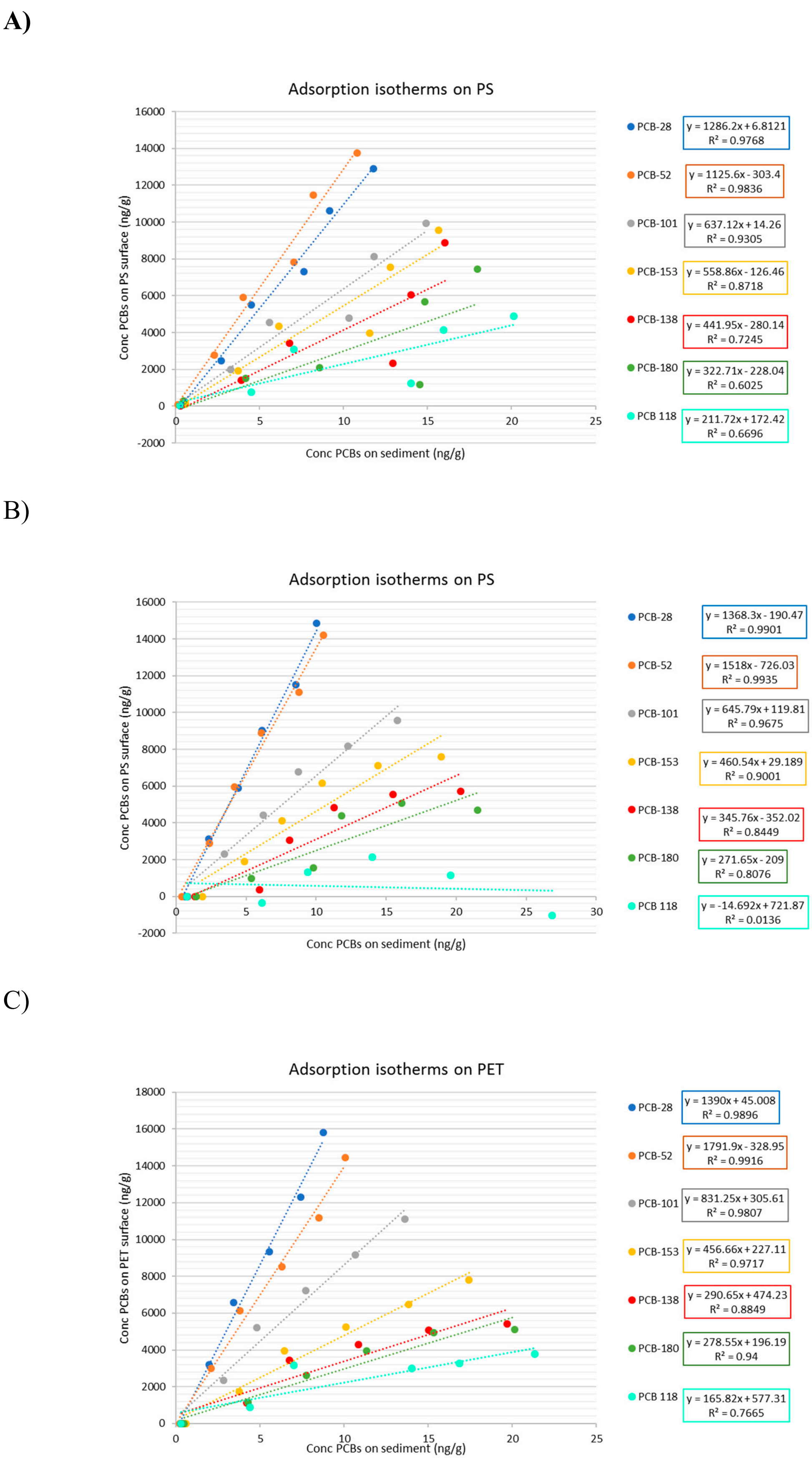

Figure S3. Individual adsorption isotherms of marker PCBs on (**A**) PS, (**B**) PE and (**C**) PET.
